# Using ROPScore and CHOP ROP for early prediction of retinopathy of prematurity in a Chinese population

**DOI:** 10.1186/s13052-021-00991-z

**Published:** 2021-02-18

**Authors:** Huiqing Sun, Yubin Dong, Yanxia Liu, Qingqin Chen, Yanxi Wang, Bin Cheng, Shaobo Qin, Liping Meng, Shanxiu Li, Yanlun Zhang, Aiguo Zhang, Weiling Yan, Yuhong Dong, Shuyi Cheng, Mingchao Li, Zengyuan Yu

**Affiliations:** 1 Department of Neonatology, Children’s Hospital of Zhengzhou University, Henan Children’s Hospital, Zhengzhou Children’s Hospital, 33 Longhuwaihuan Road, Zhengzhou, 450018 China; 2 Department of Neonatology, Zhoukou Central Hospital, Zhoukou, China; 3 Department of Neonatology, Pingdingshan People’s Hospital NO.1, Pingdingshan, China; 4 Department of Neonatology, Xinmi Maternal and Child Health Hospital, Zhengzhou, China; 5 Department of Neonatology, Zhoukou Yongshan Hospital, Zhoukou, China; 6 Department of Neonatology, Xihua People’s Hospital, Zhoukou, China; 7 Department of Neonatology, Pingyu People’s Hospital, Zhumadian, China; 8 Department of Neonatology, Jiaozuo Second People’s Hospital, Jiaozuo, China; 9 Department of Neonatology, Pingdingshan Pingmei General Hospital, Pingdingshan, China; 10 Department of Neonatology, Pingdingshan Maternal and Child Health Hospital, Pingdingshan, China; 11 Department of Neonatology, Jiyuan People’s Hospital, Jiyuan, China; 12 Department of Neonatology, Xinzheng People’s Hospital, Zhengzhou, China; 13 Department of Neonatology, Sanmenxia Central Hospital, Sanmenxia, China; 14 Department of Neonatology, Biyang People’s Hospital, Zhumadian, China

**Keywords:** Retinopathy of prematurity, Score, Preterm infant

## Abstract

**Purpose:**

Retinopathy of prematurity (ROP) is a disease that causes vision loss, vision impairment, and blindness, most frequently manifesting among preterm infants. ROPScore and CHOP ROP (Children’s Hospital of Philadelphia ROP) are similar scoring models to predict ROP using risk factors such as postnatal weight gain, birth weight (BW), and gestation age (GA). The purpose of this study was to compare the accuracy and difference between using ROPScore and CHOP ROP for the early prediction of ROP.

**Methods:**

A retrospective study was conducted from January 2009 to December 2019 in China. Patients eligible for enrollment included infants admitted to NICU at ≤32 weeks GA or those with ≤1500 g BW. The sensitivity and specificity of ROPScore and CHOP ROP were analyzed, as well as its suitability as an independent predictor of ROP.

**Results:**

Severe ROP was found in 5.0% of preterm infants. The sensitivity and specificity of the ROPScore test at any stage of ROP was 55.8 and 77.8%, respectively. For severe ROP, the sensitivity and specificity was 50 and 87.0%, respectively. The area under the receiver operating characteristic curve for the ROPScore for predicting severe ROP was 0.76. This value was significantly higher than the values for birth weight (0.60), gestational age (0.73), and duration of ventilation (0.63), when each was category measured separately. For the CHOP ROP, it correctly predicted infants who developed type 1 ROP (sensitivity, 100%, specificity, 21.4%).

**Conclusions:**

The CHOP ROP model predicted infants who developed type 1 ROP at a sensitivity of 100% whereas ROPScore had a sensitivity of 55.8%. Therefore, the CHOP ROP model is more suitable for Chinese populations than the ROPScore test.

**Clinical registration number and STROBE guidelines:**

This article was a retrospective cohort study and reported the results of the ROPScore and CHOP ROP algorithms. No results pertaining to interventions on human participants were reported. Thus, registration was not required and this study followed STROBE guidelines.

## Background

Retinopathy of prematurity (ROP) is a disease that causes vision loss, vision impairment, and blindness, most frequently manifesting among infants with low birth weight (BW) and poor health status. The survival of preterm infants has increased in the last few decades due to the rapid improvement in neonatal intensive care. Consequently, the incidence of ROP has increased, particularly in newly industrialized countries, comprising of a “third epidemic.” [1] The reported incidence of ROP that requires treatment varies from 0 to 34.8%, [2–5] depending on local neonatal care quality and characteristics of each individual patient.

The development of ROP is associated with multiple risk factors. Early gestation age (GA) and low BW are two of the most important risk factors. Other factors include blood transfusion, mechanical ventilation, anemia, respiratory distress, dyspnea, and poor health. Several screening guidelines of ROP based on GA and BW have been introduced for neonatologists to use in identification of preterm neonates who are at ≤32 weeks GA, or BW ≤1500 g. Risk criteria for preterm neonates include neonates at ≤32 weeks GA or with a BW ≤1500 g. An infant with a very unstable clinical course can also be identified to be of high risk for developing ROP, indicating a need for ophthalmology screening [6]. Challenges in identifying ROP in preterm neonates includes complying with screening guidelines, the expense of timely screenings, potential neurologic and cardiopulmonary side effects of dilated fundus examinations, and the large amount of work required by health professionals. Therefore, a more feasible methodology is necessary to identify infants who require ROP screening.

The ROPScore proposed by Eckert et al. is a scoring system that can be used to predict the severity of ROP [7]. This algorithm utilizes the following predictive variables: birth weight, gestational age, blood transfusion, mechanical ventilation and proportional weight gain at the sixth week of life. The score is calculated in the sixth week of life by use of a spreadsheet. A high score indicates that the infant has a high risk of developing severe ROP [8]. CHOP ROP (Children’s Hospital of Philadelphia ROP) used postnatal weight gain, BW, and GA in their ROP prediction model in a cohort of infants, which meets current ROP screening guidelines [9].

As far as known to the authors, only a few studies have validated this screening tool [8–11]. These studies were retrospective analyses of the efficacy of the ROPScore in American, Canadian, Italian and Brazilian populations. The purpose of this study was to evaluate the use of the ROPScore and CHOP ROP models to predict ROP in a Chinese population.

## Methods

A retrospective cohort study was conducted from January 2009 to December 2019 in NICUs in Henan Province, China. The Life Science Ethics Committee of Children’s Hospital Affiliated to Zhengzhou University approved the study (IRB number 20081227).

### Patient population

Patients eligible for enrollment included infants admitted to the NICU at GA ≤32 weeks or BW ≤1500 g. Infants with any of the following were excluded from this study: genetic metabolic diseases, congenital major abnormalities, and infants who died before the sixth week after birth.

### Weight measurements

Follow standard clinical procedures for all infants and weight measurements were conducted weekly from birth to discharge. These measurements were repeated again at a GA of 40 weeks [12].

### ROPScore screening

ROPScore Screening was conducted in the sixth week of life with a Microsoft Excel spreadsheet (Microsoft, Redmond, WA, USA), as suggested by Eckert et al. [7] This algorithm utilizes the following predictive variables: birth weight, gestational age, blood transfusion, mechanical ventilation and proportional weight gain at the sixth week of life [7]. The score is determined by linear regression, which takes into account the effect of each variable towards the onset of ROP.

### CHOP ROP screening

Binenbaum et al. developed a simpler logistic regression based model named PINT ROP [13]. The PINT ROP cohort was at a high risk for ROP. Therefore, the investigators applied the same modeling approach to a low risk cohort, which is more representative of the current US ROP screening criteria (BW < 1501 g), to develop an updated model called CHOP ROP [9]. Data was collected from medical records and entered into a web-based database, consisting of BW, GA, weight gain rate measurements, detailed demographics, ophthalmologic and medical data. Data quality was ensured through implementing data input verification rules, data review and discrepancy checking algorithms, and investigation and analysis of all tag values [11, 14].

### ROP screening and classification

ROP screening was performed for all extremely preterm infants by qualified ophthalmologists with expertise in ROP in accordance with the Chinese guidelines for the examination and treatment of ROP [15]. The choice to conduct additional ROP screening was determined according to the results of the initial screening. Termination of ROP screening was determined according to vascular development in the retina or up to 45 weeks of corrected GA [15]. ROP was subdivided into stages 1–5 based on the International Classification of ROP [16]. Mild ROP was defined as having stage 1 or stage 2 ROP in zone II or III without plus disease [12]. Type 1 ROP was defined as any stage ROP in zone I with plus disease; stage 3 ROP in zone I without plus disease; or stage 2 or 3 ROP in zone II with plus disease [17]. Type 2 ROP was defined as stage 1 or 2 ROP in zone I without plus disease; or stage 3 ROP in zone II without plus disease [17]. Severe ROP was defined as any prethreshold, any stage 3, or any threshold ROP [12].

### Clinical data collection

The following clinical data was collected: age, sex, gestational age, birth weight, number of blood transfusion, weekly weight measurements, days of mechanical ventilation and oxygen administration, ROP examination results, and the incidences of necrotizing enterocolitis (NEC), bronchopulmonary dysplasia (BPD), intraventricular hemorrhage (IVH), and sepsis. Diagnosis of ROP was conducted by pediatric ophthalmologists. Evaluations of ROP were judged as follows: none, immature, or mature vascularization. Staging of disease was performed in accordance with the International Classification of ROP [18, 19].

### Statistical analysis

SPSS software version 19.0 (SPSS, Inc., Chicago, IL, USA) was used for statistical analysis and data management. Maternal and infant characteristics were analyzed using descriptive methods and compared using t-test or one-way ANOVA (> 2 levels) for continuous variables and the chi-squared test for categorical variables. Receiver operating characteristic (ROC) curves were used to assess the accuracy of the continuous values of the ROPScore and CHOP ROP model to predict severe ROP. ROPScore was used as a dependent variable in conducting multiple linear regressions. The independent variables used in multiple logistic regression analysis were based on significant correlations and significant non-parametric univariate analyses. For severe ROP, these variables were: BW, GA, duration of ventilation, sepsis, and weight gain at the sixth week of life. The statistical significance level was set at *p* < 0.05.

## Results

### Baseline characteristics

In this study, 3624 children were screened for ROP and underwent weekly weight measurements. The ROPScore and CHOP ROP model was developed for infants with GA ≤32 weeks at birth or BW ≤1500 g. 37 infants were excluded due to incomplete weight data or because they had pathological conditions. Thus, 3587 infants born at GA ≤32 weeks or with BW ≤1500 g were included in this study. The prevalence at any stage of ROP was 372/3587 infants (10.4%). 192 preterm infants developed type 2 ROP (5.4%) and 180/3587 developed type 1 ROP that required treatment (5.0%). The baseline demographics and clinical characteristics for this cohort are shown in (Table 1). The weight gain rate was much lower in the type 1 or type 2 ROP groups compared to the group with no ROP (*p* < 0.001 respectively).

**Table 1:** Demographics of the 3587 very preterm infants included in the study

Characteristics	No ROP (*N* = 3215)	Type 2 ROP (*N* = 192)	Type 1 ROP (*N* = 180)	*P* value
Male	1938(60.2)	113(58.9)	111(61.7)	0.857
BW (g)*	1210.3 ± 217.1	1120.1 ± 225.0	1091.5 ± 221.3	< 0.001
GA (weeks)*	28.8 ± 3.7	27.9 ± 3.3	27.6 ± 3.5	< 0.001
Mean WG rate at the third week of life (g/d)*	22.1 ± 20.1	21.8 ± 15.0	18.5 ± 16.1	< 0.001
Mean WG rate at the fourth week of life (g/d)*	24.6 ± 23.1	20.6 ± 17.8	16.4 ± 12.7	< 0.001
Mean WG rate at the fifth week of life (g/d)*	19.5 ± 13.4	13.2 ± 14.6	11.2 ± 12.1	< 0.001
Mean WG rate at the Sixth week of life (g/d)*	16.8 ± 12.7	13.8 ± 14.5	13.2 ± 11.3	< 0.001

*Data are expressed as the mean ± SD; *BW* Birth Weight; *GA* Gestational Age; *ROP* Retinopathy of Prematurity; *SD* Standard Deviation; *WG* Weight gain

### ROPScore outcomes

**Fig. 1:**
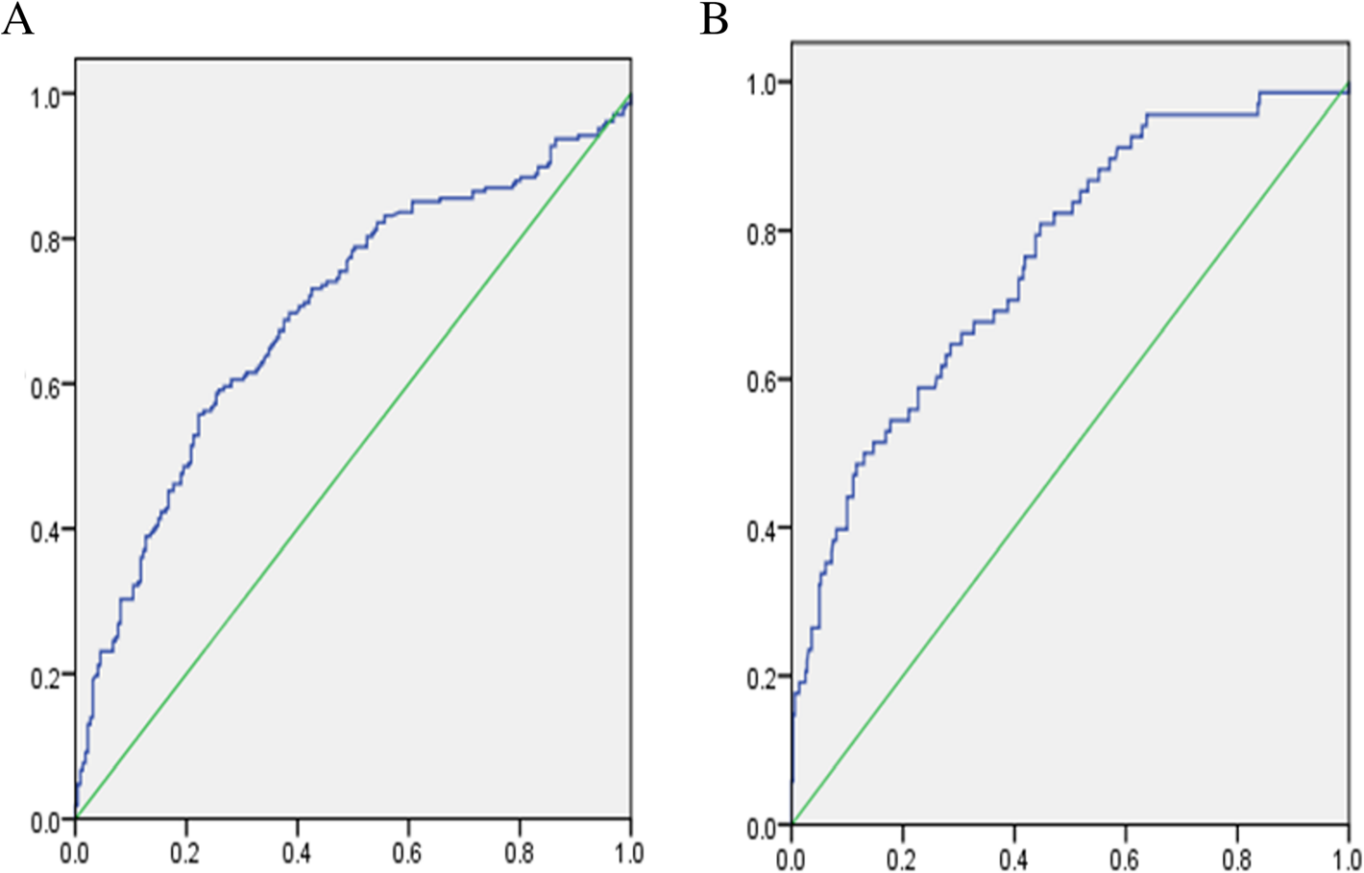
Receiver operating characteristic (ROC) curves for the detection of any stage of retinopathy of prematurity (ROP) (**a**) and of severe ROP (**b**), according to the ROPScore algorithm

The accuracy of ROPScore in predicting ROP in our participants was determined by the ROC curve (Fig. 1). Sensitivity and specificity were obtained for continuous score values by using cut-off points. The range of ROPScore values was 7.2 to 19.6. The optimal cut-off point established for any stage of ROP was 12.3 (55.8% sensitivity and 77.8% specificity), whereas the optimal cut-off point for severe ROP was 13.3 (50.0% sensitivity and 87.0% specificity).

The areas under the ROC curve for the ROPScore were 0.70 and 0.76 to predict any stage of ROP and severe ROP, respectively. The area value of severe ROP was significantly higher for ROPScore than the areas for BW (0.60), GA (0.73), and duration of ventilation (0.63), when measured separately (Table 2).

**Table 2:** Area under the ROC curve for ROPScore compared with other predictors for severe ROP

	Area under the ROC curve	*P* value	95% CI
ROPScore for severe ROP	0.76	< 0.001	0.70–0.82
BW for severe ROP	0.60	< 0.001	0.55–0.66
GA for severe ROP	0.73	< 0.001	0.67–0.78
Duration of ventilation for severe ROP	0.63	0.001	0.56–0.71
Weight gain at the sixth week of life for severe ROP	0.51	0.718	0.44–0.59
Number of blood transfusion for severe ROP	0.37	0.001	0.30–0.45

### ROPScore and infant characteristics

Multivariate logistic regression analysis showed that BW, GA, duration of ventilation, number of blood transfusions, and weight gain at the sixth week of life were risk factors for ROP. ROPScore had less tendency of predicting ROP. The unadjusted coefficient was 0.064, with an odds ratio of 1.07 at a 95% confidence interval (CI, 1.03 to 1.11). The adjusted coefficient was 1.088 with an odds ratio of 2.97 at 95% CI (0.84 to 10.45) (Table 3).

**Table 3:** multiple logistic regression analysis of the predict factors of ROPScore for severe ROP

	Undjusted Standardized Coefficients			Adjusted Standardized Coefficients		
	Beta	Odds ratio (95% CI)	*P* value	Beta	Odds ratio (95% CI)	*P* value
Birth weight	0.643	1.90(1.58–2.30)	< 0.001	0.001	1.00(1.00–1.00)	0.658
Gestational age	−0.002	1.00(1.00–1.00)	< 0.001	0.066	0.94(0.60–1.47)	0.772
Duration of ventilation	0.190	0.83(0.73–0.94)	0.003	0.021	0.98(0.91–1.05)	0.574
ROPScore	0.064	1.07(1.03–1.11)	0.001	1.088	2.97(0.84–10.45)	0.090
weight gain at sixth week of life	−0.002	1.00(1.00–1.00)	0.013	−0.001	1.00(1.00–1.00)	0.287
Number of blood transfusion	1.063	2.89(1.69–4.96)	< 0.001	1.133	0.32(0.02–4.32)	0.392

### CHOP ROP model outcomes

The infants who developed type 1 ROP were correctly predicted by the CHOP ROP model (sensitivity, 100%), but with a low specificities of 21.4% from birth to six weeks of life, 41.2% in the third week, 36.9% in the fourth week, 32.6% in the fifth week, and 38.0% in the sixth week. These results are summarized in (Table 4).

**Table 4:** Prediction of Type 1 ROP by the CHOP ROP Model Based on Birth Weight, Gestational Age, and Daily Weight Gain Rate

		Sensitivity (%)			Specificity (%)			*P value*	95% *CI*
Daily weight Gain Rate (g/d)	Area under ROC curve	0.014*	0.0026**	0.0034***	0.014	0.0026	0.0034		
From birth to six weeks of life	0.816	100%	100%	100%	21.4%	2.7%	4.3%	< 0.001	0.732 ~ 0.899
The third week	0.802	100%	100%	100%	41.2%	5.3%	9.6%	< 0.001	0.722 ~ 0.882
The fourth week	0.726	100%	100%	100%	36.9%	5.3%	7.5%	0.001	0.638 ~ 0.973
The fifth week	0.728	100%	100%	100%	32.6%	4.8%	6.4%	0.001	0.646 ~ 0.809
The sixth week	0.762	100%	100%	100%	38.0%	4.3%	8.0%	< 0.001	0.684 ~ 0.840

*CHOP ROP equation with cut point of 0.0140; **CHOP ROP equation with cut point of 0.0026; ***CHOP ROP equation with cut point of 0.0034

## Discussion

Eckert et al. developed a relatively uncomplicated model for predicting ROP in preterm infants, known as ROPScore [7]. The model is implemented using an Excel spreadsheet, which is comprised of a logistic regression equation used to calculate risk. The model includes continuous rather than dichotomized terms for BW and GA, weight gain at a single time point (6 weeks postnatal age) as a proportion of BW, dichotomous terms for blood transfusion and the use of oxygen in mechanical ventilation during the first 6 weeks of life. Assuming a specific cut-off level for low or high risk cases, ROPScore had a sensitivity of 98% and specificity of 56% for predicting ROP cases that required treatment in a cohort of 474 Brazilian infants [7]. In the present study, ROPScore had a sensitivity of 50% and specificity of 87% for predicting ROP cases that required treatment in a cohort of 3587 Chinese infants. These findings suggest that ROPScore should not be used to determine overall screening criteria. Instead, it should be used to reduce the frequency of exams in low-risk infants [7].

The poor performance of postnatal weight gain ROP models in countries with developing neonatal care systems may be related to differences in ROP pathophysiology, particularly in older GA infants. At older post-menstrual ages, endogenous production of insulin-like growth factor-1 (IGF-1) has already increased, such that low IGF-1 may play a smaller role in the pathogenesis of severe ROP [20]. In contrast, ROP in such infants might be driven primarily by high oxygen exposure, which has been shown to cause inhibition of vascular endothelial growth factor and retinal blood vessel destruction in oxygen-induced animal models of ROP. Notably, other predictive models currently undergoing testing in ROP also have limitations. For example, WINROP [21] was proposed for use in European populations and has been validated by several studies [12, 22–24], which have shown robust effectiveness in predicting ROP. However, some studies have shown that this score does not perform well in underdeveloped countries, in which moderate and late preterm infants can also develop ROP [25, 26].

We validated the CHOP ROP model in a large cohort of Chinese infants. The size of the cohort, including 180 infants who developed severe ROP, allowed us to estimate the sensitivity of the model with a high degree of precision. In this study, it was showed that the CHOP ROP model can be applied clinically to reduce the number of infants requiring examinations by one-third. No infants with type 1 ROP were excluded (sensitivity, 100%) using this model, which showed higher sensitivity compared to the evaluation of North American infants (sensitivity, 98.5%) [11]. Therefore, the CHOP ROP model could be used with confidence, ensuring that all infants with type 1 ROP are identified. The model can also be used to guide a modified screening schedule to reduce the number of examinations for lower-risk, older-GA infants.

In China, the prevalence of ROP varies according to the region, level of neonatal care, and access to ophthalmologic screening programs. Importantly, blindness caused by ROP can be prevented with timely screening [27]. The CHOP ROP and ROPScore models are useful for predicting ROP. Scoring systems have become widely used in neonatology, including neonatal intensive care, in order to aid in the detection of comorbidities. Predictive algorithms represent promising and appropriate tools that can be used to identify preterm infants at risk of developing severe ROP, as well as to reduce the excessive number of examinations performed for each preterm infant [28]. The CHOP ROP model was more sensitive than ROPScore for predicting type 1 ROP. The introduction of predictive algorithms remains in the preliminary phase and it should be emphasized that the goal is not to replace current screening guidelines. Rather, these tools can be used to help reduce the incidence of missed diagnoses of ROP [29, 30].

Regardless of the positive aspects of these predictive algorithm, there are also limitations in clinical application. First, ROPScore calculation uses preterm weight only at the sixth week of life. Hence, this test may be unable to detect high-risk preterm infants in which aggressive posterior ROP begins prior to weight measurement, then evolves rapidly [30]. Moreover, early hospital discharge of preterm infants who show robust growth is another factor that contributes to failure in collecting weight data at the correct time, which results in the inability to apply the ROPScore and CHOP ROP model.

## Conclusion

We demonstrated that the ROPScore and CHOP ROP models were an effective, promising, and noninvasive screening tool for the prediction of ROP in a Chinese population of preterm infants. The results obtained by Eckert et al. [7] were compatible with the results obtained in the present cohort regarding high sensitivity. With regard to ROPScore cut-off points, we adjusted the values for use in a Chinese population (12.3 and 13.3, for any stage of ROP and severe ROP, respectively), similar to the cut-off points used in the original study [7]. This suggests that the cut-off points would have been sufficient to detect all preterm infants with severe ROP. However, the sensitivity was lower than that reported by Eckert et al. [7] Thus, the ROPScore may need optimization for the Chinese population. The sensitivity of CHOP ROP model was higher in our study than when applied to North American infants reported by Binenbaum et al. Therefore, the CHOP ROP model may more appropriate for the Chinese population.

## Data Availability

The datasets used and/or analyzed during the current study are included in this published article.

## Abbreviations

ROP: retinopathy of prematurity
ROPScore: retinopathy of prematurity score
NICU: neonatal intensive care unit
GA: gestational age
BW: birth weight

## References

[1] Quinn GE, Gilbert C, Darlow BA, Zin A (2010). Retinopathy of prematurity: an epidemic in the making. Chin Med J.

[2] Akman I, Demirel U, Yenice O, Ilerisoy H, Kazokoglu H, Özek E (2010). Screening criteria for retinopathy of prematurity in developing countries. Eur J Ophthalmol.

[3] Dordi A., Kallen K.B.M., Ewald U.W., Jakobsson P.G., Holmstrom G.E.: Incidence of retinopathy of prematurity in infants born before 27 weeks' gestation in Sweden. Archives of Ophthalmology, 128:1289 (2011). 10.1001/archophthalmol.2009.244 19822848

[4] Palmer EA, Flynn JT, Hardy RJ, Phelps DL, Phillips CL, Schaffer DB, Tung B (1991). Incidence and early course of retinopathy of prematurity. The Cryotherapy for Retinopathy of Prematurity Cooperative Group Ophthalmology.

[5] Chiang MF, Arons RR, Flynn JT, Starren JB (2004). Incidence of retinopathy of prematurity from 1996 to 2000: analysis of a comprehensive New York state patient database. Ophthalmology.

[6] Broxterman EC, Hug DA (2016). Retinopathy of prematurity: a review of current screening guidelines and treatment options. Mo Med.

[7] Eckert G.U., Fortes Filho J.B., Maia M., Procianoy R.S.: A predictive score for retinopathy of prematurity in very low birth weight preterm infants. Eye (London, England), 26:400–406 (2012). 10.1038/eye.2011.334 PMC3298990 22193874

[8] Lucio K.C.D.V., Bentlin M.R., Augusto A.C.D.L., Corrente J.E., Toscano T.B.C., Dib R.E., Jorge E.C.: The ROPScore as a Screening Algorithm for Predicting Retinopathy of Prematurity in a Brazilian Population. Clinics, 73(2018). 10.6061/clinics/2018/e377 PMC6055020 30066729

[9] Binenbaum G., Ying G.S., Quinn G.E., Huang J., Dreiseitl S., Antigua J., Foroughi N., Abbasi S.: The CHOP postnatal weight gain, birth weight, and gestational age retinopathy of prematurity risk model. Archives of ophthalmology (Chicago, Ill : 1960), 130:1560–1565 (2012). 10.1001/archophthalmol.2012.2524 23229697

[10] Piermarocchi S, Bini S, Martini F, Berton M, Lavini A, Gusson E, Marchini G, Padovani EM, Macor S, Pignatto S (2016). Predictive algorithms for early detection of retinopathy of prematurity. Acta Ophthalmol.

[11] Binenbaum G, Ying GS, Tomlinson LA (2017). Validation of the Children's Hospital of Philadelphia retinopathy of prematurity (CHOP ROP) model. JAMA ophthalmology.

[12] Sun H, Kang W, Cheng X, Chen C, Xiong H, Guo J, Zhou C, Zhang Y, Hellström A, Löfqvist C, Zhu C (2013). The use of the WINROP screening algorithm for the prediction of retinopathy of prematurity in a Chinese population. Neonatology.

[13] Binenbaum G, Ying GS, Quinn GE, Dreiseitl S, Karp K, Roberts RS, Kirpalani H (2011). A clinical prediction model to stratify retinopathy of prematurity risk using postnatal weight gain. Pediatrics.

[14] Binenbaum G, Tomlinson LA (2017). Postnatal growth and retinopathy of prematurity study: rationale, design, and subject characteristics. Ophthalmic Epidemiol.

[15] Zhu L, Shi WJ, Zhang SL, Yu LP, Yao MZ, Shi YY, Zeng XQ, Wang SN, Chen DM, Lin ZL, Ruan FQ, Huang QW, Qian Y, Chen C (2011). Evaluation of risk factors for retinopathy of prematurity. Zhonghua Yi Xue Za Zhi.

[16] The International Classification of Retinopathy of Prematurity revisited. Archives of ophthalmology (Chicago, Ill : 1960), 123:991–999 (2005). 10.1001/archopht.123.7.991 16009843

[17] Revised indications for the treatment of retinopathy of prematurity: results of the early treatment for retinopathy of prematurity randomized trial. Archives of ophthalmology (Chicago, Ill : 1960), 121:1684–1694 (2003). 10.1001/archopht.121.12.1684 14662586

[18] Listed N. An international classification of retinopathy of prematurity. The Committee for the Classification of Retinopathy of Prematurity Archives of Ophthalmology. 1984;102(1130). 10.1001/archopht.1984.01040030908011 6547831

[19] Flynn JT (1987). An international classification of retinopathy of prematurity. Arch Ophthalmol.

[20] Darlow B.A., Binenbaum G.: Oxygen, weight gain, IGF-1 and ROP: not a straight-forward equation. Acta paediatrica (Oslo, Norway : 1992), 107:732–733 (2018). 10.1111/apa.14114 29083092

[21] Wu C., Vanderveen D.K., Hellstrom A., Lofqvist C., Smith L.E.: Longitudinal postnatal weight measurements for the prediction of retinopathy of prematurity. Archives of ophthalmology (Chicago, Ill : 1960), 128:443–447 (2010). 10.1001/archophthalmol.2010.31 PMC4393744 20385939

[22] Wirth M., Desjarlais M., Chemtob S., Hascoët J.M.: Multifactorial contributions to WINROP to enhance prediction of severe retinopathy of prematurity. Acta paediatrica (Oslo, Norway : 1992), 108:1170 (2019). 10.1111/apa.14736 30719759

[23] Sanghi G, Narang A, Narula S, Dogra MR (2018). WINROP algorithm for prediction of sight threatening retinopathy of prematurity: initial experience in Indian preterm infants. Indian J Ophthalmol.

[24] Timkovic J, Pokryvkova M, Janurova K, Barinova D, Polackova R, Masek P (2017). Evaluation of the WinROP system for identifying retinopathy of prematurity in Czech preterm infants. Biomedical papers of the Medical Faculty of the University Palacky, Olomouc, Czechoslovakia.

[25] Lofqvist C., Hansen-Pupp I., Andersson E., Holm K., Smith L.E., Ley D., Hellstrom A.: Validation of a new retinopathy of prematurity screening method monitoring longitudinal postnatal weight and insulinlike growth factor I. Archives of ophthalmology (Chicago, Ill : 1960), 127:622–627 (2009). 10.1001/archophthalmol.2009.69 19433710

[26] Perez-Munuzuri A, Fernandez-Lorenzo JR, Couce-Pico ML, Blanco-Teijeiro MJ, Fraga-Bermudez JM (2010). Serum levels of IGF1 are a useful predictor of retinopathy of prematurity. Acta Paediatr.

[27] Zin A, Gole GA (2013). Retinopathy of prematurity-incidence today. Clin Perinatol.

[28] Hutchinson AK, Melia M, Yang MB, VanderVeen DK, Wilson LB, Lambert SR (2016). Clinical models and algorithms for the prediction of retinopathy of prematurity: a report by the American Academy of ophthalmology. Ophthalmology.

[29] Lee SK, Normand C, McMillan D, Ohlsson A, Vincer M, Lyons C (2001). Evidence for changing guidelines for routine screening for retinopathy of prematurity. Archives of pediatrics & adolescent medicine.

[30] Wilkinson A.R., Haines L., Head K., Fielder A.R.: UK retinopathy of prematurity guideline. Eye (London, England), 23:2137–2139 (2009). 10.1038/eye.2008.128 18836408

